# Large tuning of the optical properties of nanoscale NdNiO_3_ via electron doping

**DOI:** 10.1515/nanoph-2025-0007

**Published:** 2025-03-28

**Authors:** Yeonghoon Jin, Teng Qu, Siddharth Kumar, Nicola Kubzdela, Cheng-Chia Tsai, Tai-De Li, Shriram Ramanathan, Nanfang Yu, Mikhail A. Kats

**Affiliations:** Department of Electrical and Computer Engineering, 5228 University of Wisconsin-Madison, Wisconsin 53706, USA; Department of Applied Physics and Applied Mathematics, Columbia University, New York 10027, USA; Department of Electrical and Computer Engineering, Rutgers, The State University of New Jersey, New Jersey 08854, USA; Nanoscience Initiative at Advanced Science Research Center (ASRC) at the Graduate Center of the City University of New York, New York 10031, USA; Department of Materials Science and Engineering, University of Wisconsin-Madison, Wisconsin 53706, USA

**Keywords:** phase-change materials, tunable optics, reconfigurable, perovskite nickelates, complex refractive index, ellipsometry

## Abstract

We synthesized crystalline films of neodymium nickel oxide (NdNiO_3_), a perovskite quantum material, switched the films from a metal phase (intrinsic) into an insulator phase (electron-doped) by field-driven lithium-ion intercalation, and characterized their structural and optical properties. Time-of-flight secondary-ion mass spectrometry (ToF-SIMS) showed that the intercalation process resulted in a gradient of the dopant concentration along the thickness direction of the films, turning the films into insulator–metal bilayers. We used variable-angle spectroscopic ellipsometry to measure the complex refractive indices of the metallic and insulating phases of NdNiO_3_. The insulator phase has a refractive index of *n* ∼ 2 and low absorption in the visible and near-infrared, and analysis of the complex refractive indices indicated that the band gap of the insulating phase is roughly 3–4 eV. Electrical control of the optical band gap, with corresponding large changes to the optical refractive indices, creates new opportunities for tunable optics.

## 1 Introduction

Phase-change materials (PCMs) with a reversible modulation of the complex refractive index – *n* (real part) and/or *κ* (imaginary part) – can enable tunable photonics applications from the visible to the terahertz [1]. Specific requirements for the optical properties of PCMs depend on the target applications, but an ideal PCM should offer large modulation of *n*, low loss (low *κ*), and fast switching speed over a wavelength range covering at least a portion of the visible or infrared (IR) [2]. The realization of such tunable optical materials would benefit many domains of optics, from spatial light modulators [3] and displays [4], to lenses with tunable focal length [5] and other tunable free-space optics [6]. While many PCMs are being actively explored, including those based on crystalline-to-amorphous transitions such as Ge-Sb-Te (GST) [7] and Ge-Sb-Se-Te (GSST) [8], and insulator-to-metal transitions such as those in VO_2_ and V_2_O_5_ [9], none have the combination of large tunability of *n* and low *κ* across a broad wavelength range in the visible and/or the near-infrared (near-IR). Therefore, there exists a need for new classes of tunable optical materials.

Perovskite rare-earth nickelates (*R*NiO_3_, where *R* is a rare-earth element such as Nd and Sm) have recently emerged as a class of PCMs that feature both temperature-driven and electron-doping-driven phase transitions. The thermal transition temperature, at which the materials change from a low-temperature insulating state to a high-temperature metallic state, depends on *R*, for example ∼200 K for NdNiO_3_ and ∼400 K for SmNiO_3_ [10], [11]. However, these materials have substantial optical losses in both metallic (high-temperature) and semiconducting (low-temperature, with a small gap of the order of 0.1 eV) phases [12], and therefore applications of the thermally driven transition are limited; for example, SmNiO_3_ has been used for dynamic tuning of thermal radiation [13], but is less suited for phase modulation in a waveguide or a resonant structure.

New opportunities for *R*NiO_3_ have opened up with experiments demonstrating large and reversible tuning of the band gap via electric-field-driven electron doping at room temperature, achieved through the intercalation of H^+^ or Li^+^ ions [14]. First-principles theoretical calculations suggest that band gaps of ∼3 eV can be opened via electron doping regardless of the ionic species used for intercalation [15], [16]. Experiments have demonstrated a change in electrical resistivity of 7–8 orders of magnitude [14], [17] and a transition from an optically lossy state to a low-loss state over a broad wavelength range from the visible to the IR [18]. Note that the doping-driven phase transition gives rise to a much larger change of material properties compared to those achievable using the temperature-driven phase transition [11], [14], and that the electron-doping tuning is non-thermal, non-volatile, and controllable via electric fields [18], [19], [20].

While several studies exist on the electrical resistivity changes due to electron doping, there have been very few studies on the optical properties of the intrinsic and electron-doped states of *R*NiO_3_. A few experimental studies measured the complex refractive indices of intrinsic and electron-doped SmNiO_3_ [14], [18], with the assumption that electron-doped SmNiO_3_ films were uniformly doped. Several *ab-initio* studies have predicted the band gap of H^+^- or Li^+^-doped *R*NiO_3_ [15], [16], but the results range from 0.5 to 5 eV, depending on the calculation method. As a result, there is a need for accurate measurement of the doping profile and the optical properties of electron-doped *R*NiO_3_. The compound NdNiO_3_ is of particular interest because NdNiO_3_ can be grown at the ambient atmosphere (∼1 bar), whereas EuNiO_3_ and SmNiO_3_ generally requires high oxygen pressures (∼100 bar) [13]. Furthermore, pristine NdNiO_3_ is metallic at room temperature, whereas SmNiO_3_ is semiconducting with a bandgap of a few hundred meV [14], so we expect NdNiO_3_ to exhibit greater tuning of its optical and electrical properties than SmNiO_3_ across the phase transition.

Here, we measured the complex refractive indices of both undoped (metallic) and Li^+^-doped (insulating) NdNiO_3_ films using variable-angle spectroscopic ellipsometry (VASE) over a broad wavelength range from the ultraviolet to the near-IR (*λ* = 0.3–2.5 µm). The films were electron-doped via intercalation of lithium (Li) ions using a liquid electrolyte from their top surfaces, and the doping process resulted in a non-uniform doping profile in the depth direction of the films, as characterized by time-of-flight secondary-ion mass spectrometry (ToF-SIMS). The films that are composed of a doped top layer and an under-doped bottom layer were characterized by VASE using a two-layer model. The results showed dramatically different complex refractive indices between the two phases, with the extinction coefficient (*i.e.*, imaginary part of the complex refractive index) *κ* of Li^+^-doped NdNiO_3_ below 0.1 in the visible (*λ* = 0.4–0.7 µm) and below 0.02 in the near-IR (*λ* = 0.7–2.5 µm). Using these optical data, we estimated the band gap of the Li^+^-doped NdNiO_3_ to be roughly 3–4 eV.

## 2 Material preparation and optical experiments

### 2.1 NdNiO_3_ on electrically conductive, Nb-doped SrTiO_3_

**Figure 1:**
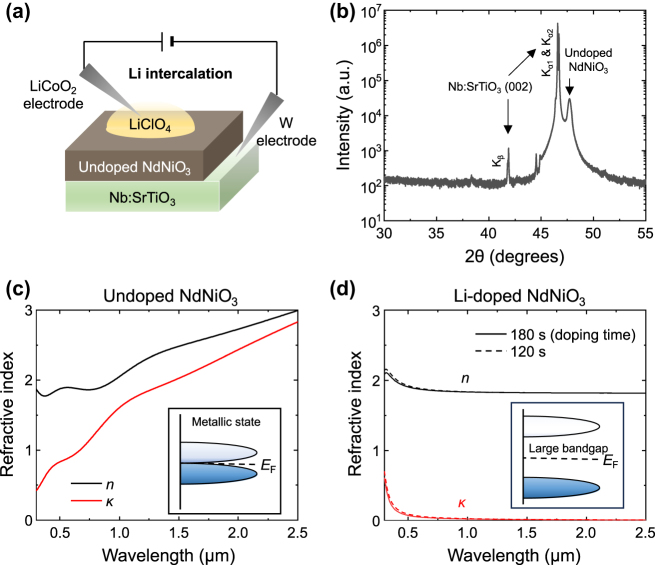
Structural and optical properties of the undoped and Li^+^-doped NdNiO_3_ thin films grown on Nb:STO substrates. (a) Depiction of the Li intercalation experiment. (b) X-ray diffraction (XRD) measurement of an undoped NdNiO_3_/Nb:STO sample. (c,d) Measured complex refractive indices (*n* and *κ*) of (c) the undoped NdNiO_3_, which has no band gap at room temperature, and (d) the Li^+^-doped NdNiO_3_ films. The curves in (d) represent the complex refractive indices of the Li^+^-doped films with different doping times of 120 s and 180 s (refer to Figure S1 for sample description).

NdNiO_3_ crystalline thin films 50 nm in thickness were grown on 0.5 % Nb-doped SrTiO_3_ (Nb:STO) substrates via high-vacuum reactive magnetron sputtering at room temperature, as shown in Figure 1a. This substrate was chosen because of the small lattice mismatch with NdNiO_3_. Two separate targets for Nd (125 W RF power) and Ni (75 W DC power) were co-sputtered in a mixed argon–oxygen (40:10 sccm) atmosphere maintained at 5 mTorr. During growth, the substrate holder was rotated at 25 rpm to ensure uniform growth. After deposition, the films were annealed at 500 °C in ambient atmosphere for 24 h to achieve full oxidation of the films to the desired phase. The X-ray diffraction (XRD) measurement of the undoped NdNiO_3_ on Nb:STO, shown in Figure 1b, confirms the small lattice mismatch and the epitaxial growth of NdNiO_3_: the two adjacent peaks around 46.6° are from the crystalline Nb:STO substrate, and the single peak at 47.7° is from the undoped crystalline NdNiO_3_ thin film; these peaks are very close to each other. Note that the Cu source used for the XRD measurement contains Cu Kα_1_, Kα_2_, and K_β_ wavelengths, showing three peaks for Nb:STO.

To trigger the metal-to-insulator transition, a droplet of an electrolyte containing Li ions (0.1 mol saturated solution of LiClO_4_ dissolved in propylene carbonate) was placed on the top of the film. Then, a top electrode (a LiCoO_2_-coated aluminum foil) was dipped into the electrolyte, the other tungsten electrode was connected to the exposed bottom Nb:STO substrate (Figure 1a), and a positive voltage of 5.5 V was applied across the electrodes to drive Li ions into the NdNiO_3_ thin film from the top electrode, while electrons were simultaneously driven into the thin film via the bottom electrode. The samples were rinsed with anhydrous ethanol, deionized water (with sonication), and isopropanol, followed by drying with nitrogen gas flow. The complex refractive index (*n* and *κ*) of an undoped NdNiO_3_ film is shown in Figure 1c, and that of Li^+^-doped NdNiO_3_ films with different doping times (120, and 180 s) is shown in Figure 1d (detailed explanation on how these results were obtained will be described in the next section). The complex refractive index of the undoped film is indicative of a metal, with *κ* increasing as a function of wavelength, while that of the Li^+^-doped films is indicative of an insulator possessing a large band gap, with small *κ* decreasing with increasing wavelength. The average *κ* in the visible (*λ* = 0.4–0.7 µm) for the Li^+^-doped state is lower than 0.1, and it decreases further in the near-IR.

### 2.2 Spectroscopic ellipsometry and ToF-SIMS analysis

**Figure 2:**
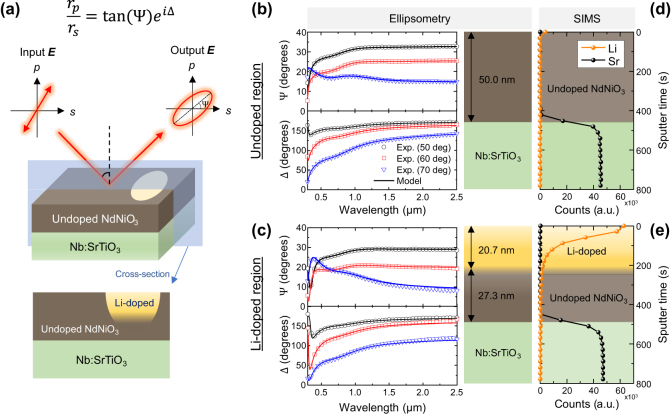
Spectroscopic ellipsometry and ToF-SIMS analysis of an NdNiO_3_/Nb:STO sample. (a) Schematic of the ellipsometric measurement. There is a Li^+^-doped region with a gradient Li ion concentration profile in the depth direction. (b,c) Ellipsometric parameters Ψ and Δ measured on (b) the undoped NdNiO_3_ region and (c) the Li^+^-doped NdNiO_3_ region, at three angles of incidence of 50°, 60° and 70° (symbols). The solid curves represent a model fit, assuming one layer in the case of the undoped region and two layers for the doped region. The right panel depicts the estimated thickness of each layer based on ellipsometry. (d,e) Depth-dependent Li and Sr counts of (d) the undoped region and (e) the Li^+^-doped region, measured by ToF-SIMS.

Figure 2a shows the process by which we extracted the complex refractive indices (shown in Figure 1c and d) for both the undoped and Li^+^-doped NdNiO_3_ regions. We used a VASE ellipsometer (J.A. Woollam Co.) in the wavelength range from 0.3 to 2.5 µm to measure the depolarization of light when it reflects from a NdNiO_3_ film on an Nb:STO substrate. The output of a VASE measurement for a given angle of incidence are spectra of Ψ and Δ, defined as tan(Ψ)*e* ^*i*Δ^ = *r*_*p*_/*r*_*s*_, where *r*_*p*_ and *r*_*s*_ are the complex-valued reflection coefficients for *p*- and *s*-polarized light, respectively [21]. By fitting the experimentally obtained wavelength-dependent Ψ and Δ with a model, we can determine the complex refractive index and thickness of the NdNiO_3_ film.

We first measured the complex refractive index of the substrate (Nb:STO); see Figure S2 for the ellipsometry measurements and resulting optical properties of Nb:STO. Figure 2b shows the parameters Ψ and Δ of the undoped NdNiO_3_ region from *λ* = 0.3–2.5 µm, at three angles of incidence (50°, 60°, and 70°). Note that a focusing probe was used to ensure that the beam spot was much smaller than the measured undoped NdNiO_3_ region. The solid curves represent fitting of the experimental results using a single-layer model, showing good agreement between the model and the experiment. The NdNiO_3_ complex refractive index is modeled using one Drude and three Lorentz oscillators (see Note S1 in the Supporting information for detailed description of the model). The fitted complex refractive index of undoped NdNiO_3_ is shown in Figure 1c and the fitted thickness is 50 nm.

Figure 2c shows the parameters Ψ and Δ of the Li^+^-doped region (doping time of 180 s) from *λ* = 0.3–2.5 µm, at three angles of incidence (50°, 60°, and 70°). We were not able to fit the experimental Ψ and Δ spectra with a single-layer model, suggesting that the doped NdNiO_3_ region is not uniform through its thickness. Because the source of Li ions was on the top surface of the film, it is reasonable to assume a gradient of the Li ion concentration in the depth direction. Thus, we used a two-layer model to fit the ellipsometry results, assuming that there is a fully doped layer on the top and an undoped layer on the bottom (lower panel in Figure 2a). Our two-layer model shows good agreement with the experimental Ψ and Δ (Figure 2c). The estimated thickness of the Li^+^-doped NdNiO_3_ layer is 20.7 nm and that of the undoped layer is 27.3 nm; the total thickness of 20.7 + 27.3 = 48 nm is close to the thickness measured in the undoped region of 50 nm. See Note S1 for a detailed description of the two-layer model, the fitting process, and the oscillator parameters.

To validate our ellipsometry fitting model, we conducted ToF-SIMS measurements to characterize the vertical doping profile of Li ions (see Note S2 for a description of the measurement conditions). Figure 2e shows the ToF-SIMS profile of Li and Sr, measured on the Li^+^-doped region, and the same measurement was conducted on the undoped region for comparison (Figure 2d). The results show that the Li ion concentration is negligible in the undoped region, whereas in the Li^+^-doped region the Li ion concentration exponentially decays from the top surface. A recent theoretical study [22] has shown that the band gap rapidly opens up when the full electron doping level of one ion and one electron per Ni is approached (*e.g.*, in H^+^-doped SmNiO_3_, the band gap is only 1.4 eV at the half doping level of H^+^:Ni = 0.5:1, but abruptly increases to 5.1 eV at the full doping level of H^+^:Ni = 1:1). This justifies the two-layer model as an approximation of a partially doped thin film, where we assume that the fully doped and over-doped top layer has a fully opened band gap represented by *n* and *κ* characteristic of an insulator, and the undoped and insufficiently doped bottom layer has minimal band gap opening represented by *n* and *κ* that can be approximated by the complex refractive index of the undoped NdNiO_3_.

### 2.3 NdNiO_3_ on transparent LaAlO_3_

**Figure 3:**
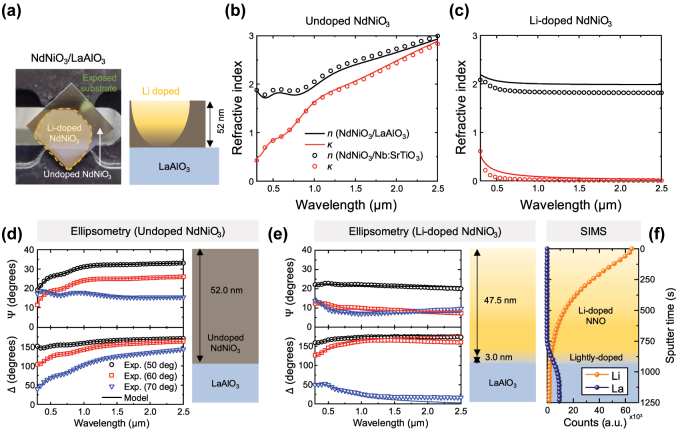
Measurement of undoped and Li^+^-doped NdNiO_3_ thin films grown on LaAlO_3_ (LAO) substrates. (a) A photo of our NdNiO_3_/LAO sample, including the Li^+^-doped region, the undoped region, and the exposed substrate region. (b,c) Extracted complex refractive indices of (b) the undoped NdNiO_3_ and (c) the Li^+^-doped NdNiO_3_ (curves). The complex refractive indices of NdNiO_3_ on Nb:STO (extracted from Figure 2b and c) are also shown for comparison (symbols). (d,e) The Ψ and Δ data of (d) the undoped NdNiO_3_ and (e) the Li^+^-doped NdNiO_3_, at three angles of incidence (50°, 60°, and 70°). The solid curves represent a model fit. The right panel shows the thickness estimated by ellipsometry. (f) ToF-SIMS results of the doped NdNiO_3_ region, showing the Li and La concentration depth profiles.

We have also grown NdNiO_3_ thin films on crystalline LaAlO_3_ (LAO) substrates. This substrate was chosen because it provides a nearly lattice matched platform for epitaxial growth of crystalline NdNiO_3_ and allows the change in transparency of Li^+^-doped NdNiO_3_ regions to be readily observed because LAO is transparent (unlike Nb:STO, which is opaque in the visible spectrum). Refer to Figure S3 for X-ray diffraction (XRD) and Rutherford backscattering (RBS) characterization of the NdNiO_3_/LAO sample. Since the LAO substrate is not electrically conductive, the voltage for Li intercalation of NdNiO_3_ was applied between the top electrode in the electrolyte (as shown in Figure 1a) and a bottom electrode placed on undoped NdNiO_3_, with 5.5 V applied across the electrodes for 10 min. Figure 3a shows a photo of the sample after the Li intercalation experiment: the large transparent region is where NdNiO_3_ is doped with Li ions, the dark region represents undoped NdNiO_3_, and the smallest region is the exposed LAO substrate, from where the sample was mounted using a clip in the sputtering chamber. The doped region is nearly as transparent as the exposed substrate, suggesting low *κ* of the doped NdNiO_3_ in the visible.

VASE and ToF-SIMS were used to characterize the NdNiO_3_/LAO samples as well as the LAO substrate. The ellipsometry data and the refractive index of the LAO substrate are shown in Figure S4. The refractive index of the undoped NdNiO_3_ region on LAO are shown in Figure 3b and that of the Li^+^-doped NdNiO_3_ region are shown in Figure 3c, both very similar to the measurements of the NdNiO_3_ films on Nb:STO. The thickness of the undoped NdNiO_3_ film on LAO extracted from VASE is 52 nm (Figure 3d). For the doped region, the two-layer model shows good fitting with the experimental data (Figure 3e), and the estimated thickness of the top layer is 47.5 nm and that of the bottom layer is only 3 nm (refer to Note S1 for a detailed description of the fitting process). ToF-SIMS independently confirmed that Li ions are present almost over the entire thickness of the NdNiO_3_ film with a gradient concentration profile (Figure 3f). The deeper Li ion penetration depth for the NdNiO_3_/LAO sample results from a longer Li intercalation time (600 s) compared to that of the NdNiO_3_/Nb:STO sample in Figure 2e (60–180 s).

### 2.4 NdNiO_3_ on transparent conducting oxides

Because NdNiO_3_ can be grown at ambient pressure – while EuNiO_3_ and SmNiO_3_ requires high oxygen pressures (∼100 bar) – it can be deposited more readily on various substrates, including indium-tin-oxides (ITO) and fluorine-doped tin-oxides (FTO), which are widely studied transparent conductors. Refer to Figure S5 for images of Li-doped NdNiO_3_ on ITO or FTO, and Figure S6 for the optical reversibility test of a NdNiO_3_/ITO glass structure.

One can choose and optimize the substrate, thickness, and doping condition depending on the target applications. For example, for electrochromic windows, a 100-nm-thick NdNiO_3_ on ITO or FTO would be suitable, as the ITO/FTO substrate is transparent, and a thickness of 100 nm enables transmittance modulation between fully opaque and transparent states (Figure S5).

## 3 Estimation of the optical band gap

A few theoretical papers have made first-principles predictions of the band gap of H^+^- or Li^+^-doped *R*NiO_3_ [15], [16], but the predicted band gaps vary over a wide range due to the assumptions used in the calculations. Here, we estimated the band gap of Li^+^-doped NdNiO_3_ using the Tauc plot method. The Tauc plot equation is given by: (*αhv*)^*m*^ = *A*(*hv*−*E*_*g*_), where *α* is the absorption coefficient, *h* is the Planck constant, *v* is the frequency of incident light, *A* is a constant, and *E*_*g*_ is the optical band gap. Extrapolating the linear region of the Tauc plot to the horizontal axis yields the band gap [23]. The power, *m*, is 0.5 for indirect transitions and 2 for direct transitions [23].

**Figure 4:**
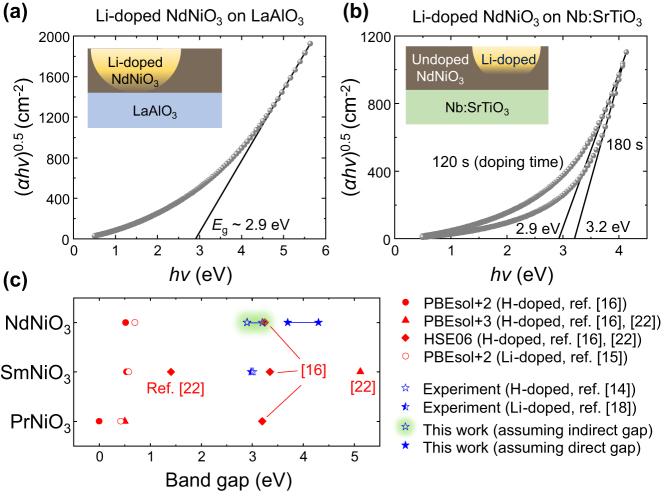
Tauc plots to estimate the optical band gap of doped NdNiO_3_, assuming that the gap is indirect. (a) Tauc plot for the Li^+^-doped NdNiO_3_ film on LAO, calculated from the data in Figure 3c. (b) The same plot for the Li^+^-doped NdNiO_3_ film on Nb:STO, calculated from the data in Figure 1d. Based on these plots, the estimated band gap is roughly 3–4 eV. (c) Theoretically and experimentally estimated band gaps of H^+^- or Li^+^-doped PrNiO_3_, SmNiO_3_, and NdNiO_3_ from the literature [14], [15], [16], [18], [22] and from our experiments, with two assumptions: (i) assuming the gap is indirect, as in (a, b), and (ii) assuming that the gap is direct, as shown in Figure S6.

Figure 4a and b shows the Tauc plots of the Li^+^-doped NdNiO_3_ films on LAO and Nb:STO, respectively, assuming an indirect gap. The estimated band gap is 2.9–3.2 eV, matching some of the theoretical predictions [16] and experimental reports on H^+^- or Li^+^-doped SmNiO_3_ [14], [18]. Assuming that the gap is direct leads to an estimated gap of 3.7–4.3 eV (Figure S7). Figure 4c shows the theoretically [15], [16], [22] and experimentally [14], [18] estimated band gaps of H^+^- or Li^+^-doped PrNiO_3_, SmNiO_3_, and NdNiO_3_, including our experiments. The band gap of H^+^- or Li^+^-doped *R*NiO_3_ estimated by first-principles calculations varies from ∼0 to ∼5.1 eV depending on the underlying assumptions. A number of these theoretical results are aligned with our experimental estimates, which estimate the band gap to be roughly 3–4 eV. We would expect that the band gap of most H- or Li-doped *R*NiO_3_ would be on this order, given that previous theoretical predictions [15], [16] show H- or Li-*R*NiO_3_ as having similar band gaps.

We note that a recent paper reported that the Tauc plot method may not be applicable to Mott-transition materials such as Co_3_O_4_ and Co_x_Fe_3-x_O_4_ because the complex bands permit many optical transitions so it is not possible to determine which absorption is originated from the valence band maximum to the conduction band minimum [23]. However, unlike Co_3_O_4_ and Co_x_Fe_3-x_O_4_, the optical absorption of our Li^+^-doped NdNiO_3_ is substantial only at wavelengths shorter than 400 nm (Figure 1d), which suggests that the absorption is caused by the band edge-to-edge transition. Thus, we expect that the Tauc plot can still be used to estimate the band gap of H^+^- or Li^+^-doped *R*NiO_3_.

## 4 Conclusions

We characterized, for the first time to our knowledge, the complex refractive indices of both undoped and Li^+^-doped NdNiO_3_ films at wavelengths from 0.3 to 2.5 µm. Upon electron doping via Li intercalation, NdNiO_3_ undergoes a phase transition, where the band gap changes from near-zero (metallic state) to 3–4 eV (insulating state). In our samples, the Li ion concentration profile in the NdNiO_3_ films has a gradient in the depth direction, as evidenced by ToF-SIMS; thus, we introduced a two-layer model to fit data from VASE. The results show that the extinction coefficient (*κ*) of the insulating state of NdNiO_3_ is lower than 0.1 in the visible and 0.02 in the near-IR. Future efforts to improve the performance of the rare-earth perovskite nickelates will focus on precisely controlling the electron doping levels via Li or proton intercalation so that we can leverage the large optical refractive index dispersion in proximity to the band edge to realize large and continuous refractive index tuning and low optical losses across the visible and near-IR ranges. Given the broad interest of tuning physical properties of quantum materials via electric-field gating, the method presented here to analyze nanoscale matter with inhomogeneous electron concentrations and the large tuning of optical properties should open new directions to design electrically tunable solids.

## Supplementary Material

Supplementary Material Details

## References

[1] Ko J. H., Yoo Y. J., Lee Y., Jeong H.-H., Song Y. M. (2022). A review of tunable photonics: Optically active materials and applications from visible to terahertz. *IScience*.

[2] Veeder T. (2024). Accelerating discovery of tunable optical materials (ATOM). *Image Sens. Technol.: Mater., Devices, Syst., Appl. XI*.

[3] Li S.-Q., Xu X., Maruthiyodan Veetil R., Valuckas V., Paniagua-Domínguez R., Kuznetsov A. I. (2019). Phase-only transmissive spatial light modulator based on tunable dielectric metasurface. *Science*.

[4] Hosseini P., Wright C. D., Bhaskaran H. (2014). An optoelectronic framework enabled by low-dimensional phase-change films. *Nature*.

[5] Wang Q. (2016). Optically reconfigurable metasurfaces and photonic devices based on phase change materials. *Nat. Photonics*.

[6] Julian M. N., Williams C., Borg S., Bartram S., Kim H. J. (2020). Reversible optical tuning of GeSbTe phase-change metasurface spectral filters for mid-wave infrared imaging. *Optica*.

[7] Wuttig M., Bhaskaran H., Taubner T. (2017). Phase-change materials for non-volatile photonic applications. *Nat. Photonics*.

[8] Zhang Y. (2019). Broadband transparent optical phase change materials for high-performance nonvolatile photonics. *Nat. Commun.*.

[9] Ko B., Badloe T., Rho J. (2021). Vanadium dioxide for dynamically tunable photonics. *Chem. Nano. Mat.*.

[10] Lacorre P., Torrance J., Pannetier J., Nazzal A., Wang P., Huang T. (1991). Synthesis, crystal structure, and properties of metallic PrNiO_3_: Comparison with metallic NdNiO_3_ and semiconducting SmNiO_3_. *J. Solid State Chem.*.

[11] Torrance J., Lacorre P., Nazzal A., Ansaldo E., Niedermayer C. (1992). Systematic study of insulator-metal transitions in perovskites R NiO_3_ (R= Pr, Nd, Sm, Eu) due to closing of charge-transfer gap. *Phys. Rev. B*.

[12] Stewart M., Liu J., Kareev M., Chakhalian J., Basov D. (2011). Mott physics near the insulator-to-metal transition in NdNiO_3_. *Phys. Rev. Lett.*.

[13] Shahsafi A. (2019). Temperature-independent thermal radiation. *Proc. Natl. Acad. Sci. U. S. A.*.

[14] Shi J., Zhou Y., Ramanathan S. (2014). Colossal resistance switching and band gap modulation in a perovskite nickelate by electron doping. *Nat. Commun.*.

[15] Cui Y., Liu X., Fan W., Ren J., Gao Y. (2021). Metal–insulator transition in RNiO_3_ (R= Pr, Nd, Sm, Gd, Tb, Dy, Ho, Er) induced by Li doping: A first-principles study. *J. Appl. Phys.*.

[16] Yoo P., Liao P. (2020). First principles study on hydrogen doping induced metal-to-insulator transition in rare earth nickelates RNiO_3_ (R= Pr, Nd, Sm, Eu, Gd, Tb, Dy, Yb). *Phys. Chem. Chem. Phys.*.

[17] Sun Y. (2018). Strongly correlated perovskite lithium ion shuttles. *Proc. Natl. Acad. Sci. U. S. A.*.

[18] Li Z. (2016). Correlated perovskites as a new platform for super-broadband-tunable photonics. *Adv. Mater.*.

[19] Shi J., Ha S. D., Zhou Y., Schoofs F., Ramanathan S. (2013). A correlated nickelate synaptic transistor. *Nat. Commun.*.

[20] Zhang H.-T. (2022). Reconfigurable perovskite nickelate electronics for artificial intelligence. *Science*.

[21] Fujiwara H., Collins R. W. (2018). *Spectroscopic Ellipsometry for Photovoltaics*.

[22] Yamauchi K., Hamada I. (2023). Hydrogen-induced insulating state accompanied by interlayer charge ordering in SmNiO3. *Phys. Rev. B*.

[23] Klein J., Kampermann L., Mockenhaupt B., Behrens M., Strunk J., Bacher G. (2023). Limitations of the Tauc plot method. *Adv. Funct. Mater.*.

