# Supplementary material for: Large tuning of the optical properties of nanoscale NdNiO3 via electron doping
Source: Nanophotonics. 2025 Mar 28;14(23):4125–32. doi: 10.1515/nanoph-2025-0007 (PMC12617736; doi:10.1515/nanoph-2025-0007)
Supplement: Supplementary file 1 — Supplementary Material Details [file j_nanoph-2025-0007_suppl_001.pdf]

Supporting information:

Large tuning of the optical properties of nanoscale $NdNiO_3$ via electron doping

Yeonghoon Jin[1,#], Teng Qu[2,#], Siddharth Kumar[3,#], Nicola Kubzdela[2], Cheng-Chia Tsai[2], Tai-De Li[4], Shriram Ramanathan[3,*], Nanfang Yu[2,*], Mikhail A. Kats[1,5,*]

[1]Department of Electrical and Computer Engineering, University of Wisconsin-Madison, Wisconsin 53706, USA

[2]Department of Applied Physics and Applied Mathematics, Columbia University, New York 10027, USA

[3]Department of Electrical and Computer Engineering, Rutgers, The State University of New Jersey, New Jersey 08854, USA

[4]Nanoscience Initiative at Advanced Science Research Center (ASRC) at the Graduate Center of the City University of New York, New York 10031, USA

[5]Department of Material Science and Engineering, University of Wisconsin-Madison, Wisconsin 53706, USA

[#]These authors contributed equally.

*Corresponding authors: Mikhail A. Kats (mkats@wisc.edu), Shriram Ramanathan (shriram.ramanathan@rutgers.edu), Nanfang Yu (ny2214@columbia.edu)

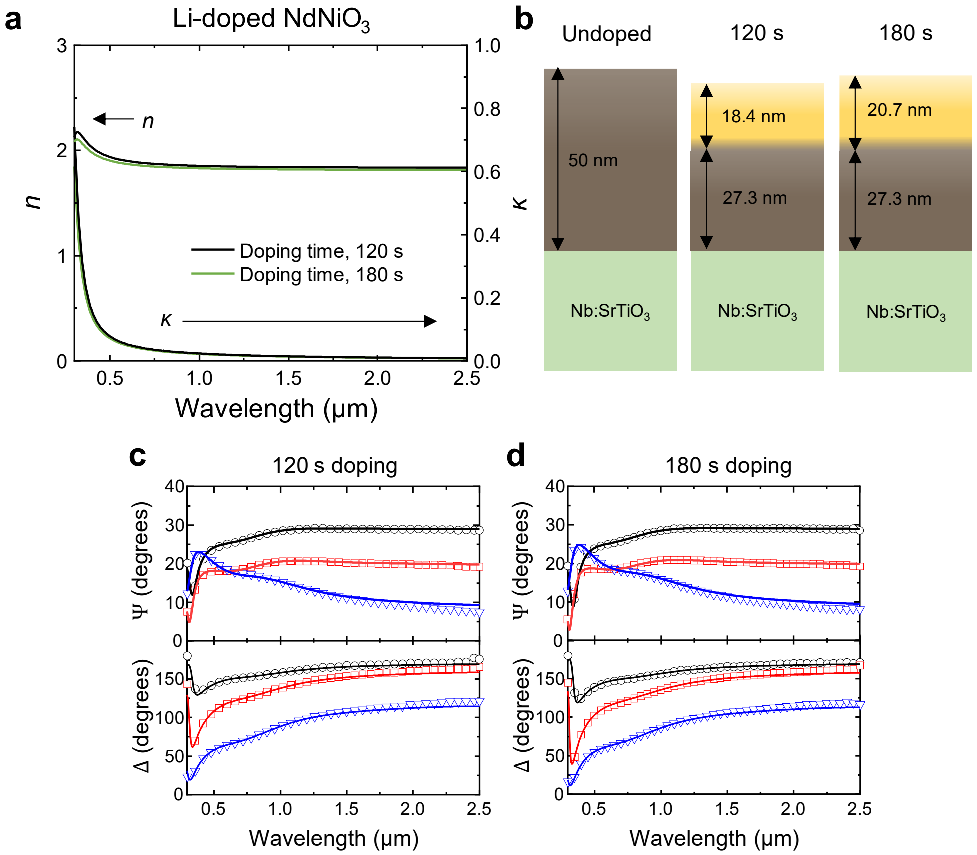

Figure S1. Optical properties of two Li-doped $NdNiO_3$ films on Nb:STO (0.5% Nb-doped $SrTiO_3$) with different doping times. **(a)** Complex refractive index of the Li-doped $NdNiO_3$ with doping times of 120 s and 180 s. **(b)** Illustrations of the samples with the thickness estimated by ellipsometry. Doping was conducted on one chip but at different spots, and therefore the entire film thickness of each spot should be the same as in the undoped region (50 nm). However, the film thicknesses of the doped spots are slightly off from 51 nm, and this discrepancy might be because we used a two-layer model (the top doped and bottom undoped layers) without considering intermediate doping states between the top and bottom layers. Nevertheless, the independently obtained refractive indices of the Li-doped $NdNiO_3$ are consistent with each other, as shown in (a), and we believe the results are reliable. **(c-e)** Ellipsometry results (Ψ and Δ) of the two spots with different doping time. See **Note S1** for detailed description for the oscillators used to fit the experiments.

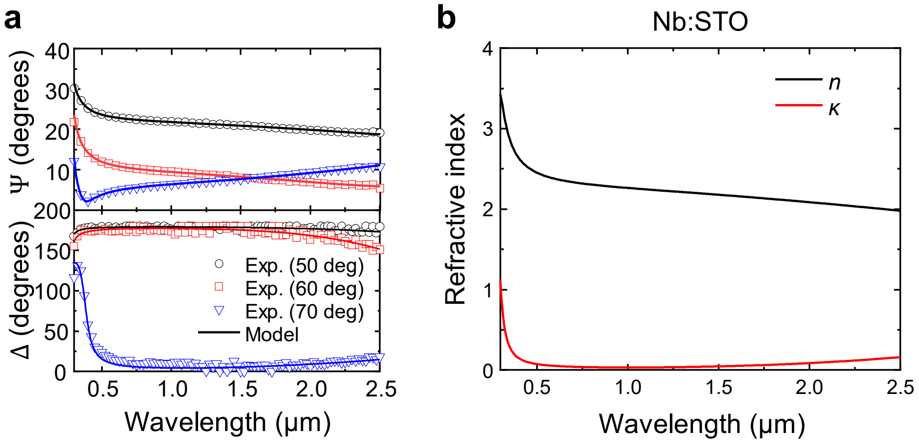

Figure S2. Optical properties of 0.5% Nb-doped $SrTiO_3$ (Nb:STO). **(a)** Spectroscopic ellipsometry results (Ψ and Δ) at three angles of incidence (50, 60, and 70 degrees) with the model fits. The oscillators used in the model are summarized in **Table S1**. **(b)** Complex refractive index of Nb:STO.

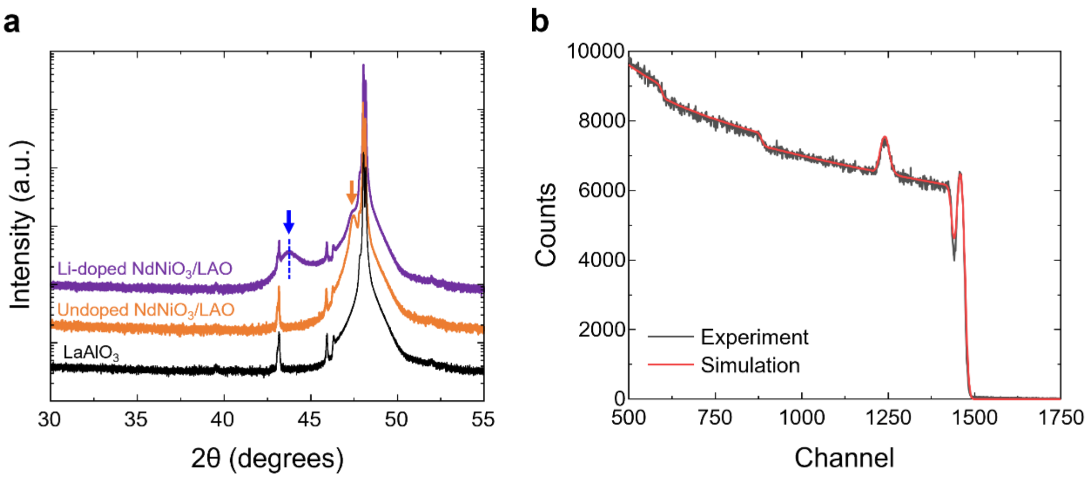

Figure S3. Material characterization for $NdNiO_3$ film on $LaAlO_3$ (LAO). **(a)** X-ray diffraction (XRD) data for a LAO substrate and both the undoped and Li-doped $NdNiO_3$ regions of the same sample. The two strongest adjacent peaks around 48.1° represents the LAO substrate. The peak at 47.5° (marked by the orange arrow) represents the undoped $NdNiO_3$; the $NdNiO_3$ and LAO peaks are very close each other, indicating the small lattice mismatch. After Li doping, a reduced intensity of the undoped $NdNiO_3$ peak is observed, along with the appearance of a feature at lower 2θ values (at 43.7°, indicated by the blue arrow). This is very similar to the case of H-doped $NdNiO_3$, where the absorbed H-ions get bonded to the O-ions in NiO_6 octahedra, increasing the lattice constant and opening a bandgap because of electron transfer.[1] **(b)** Rutherford backscattering spectroscopy (RBS) data for the undoped $NdNiO_3$/LAO, obtained by bombarding a 2-mm-wide 2.3 MeV He^{2+} ion beam and observing the backscattered particles. The x-axis (channels) represents the energy of the backscattered particles: the higher the channel number, the heavier the particle. The simulation (the red line) was conducted by assuming the $NdNiO_3$ film composition of Nd:Ni:O =

22:18:60 and the film thickness of 38 nm, and it shows good agreement with the experiment. This confirms that the film is uniform throughout its thickness.

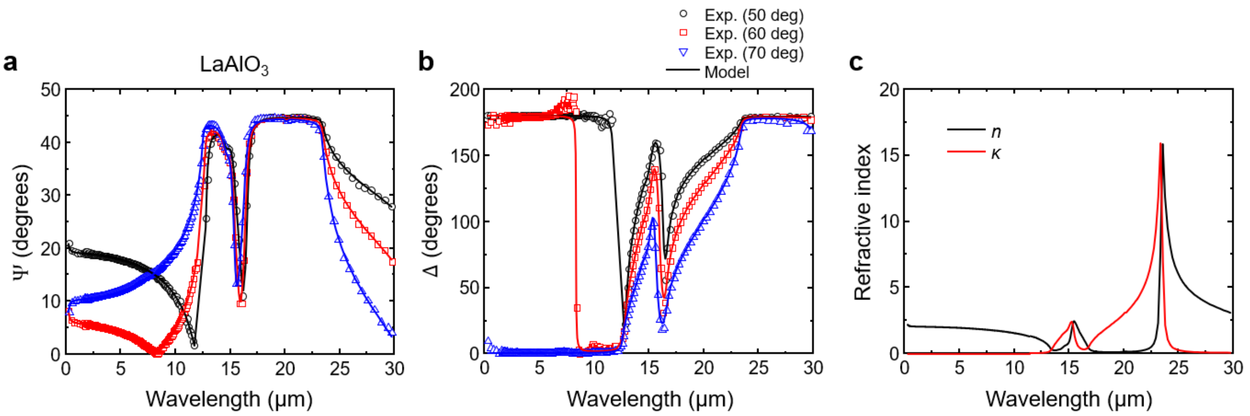

Figure S4. Optical properties of $LaAlO_3$ (LAO). **(a)** Spectroscopic ellipsometry results (Ψ and Δ) at three angles of incidence (50, 60, and 70 degrees) with the model fits. The oscillators used in the model are summarized in **Table S1**. **(b)** Complex refractive index of LAO.

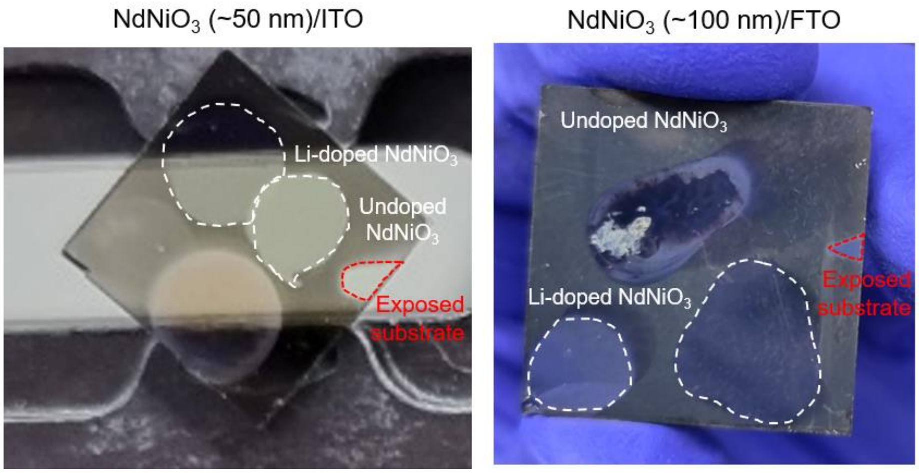

Figure S5. Photos of $NdNiO_3$ on ITO (left) and FTO (right). The substrate is covered with an $NdNiO_3$ film, except for the exposed substrate region (marked in red). Droplets of electrolyte were placed on the undoped $NdNiO_3$, and Li intercalation was performed (marked in white). The color and transparency of the most Li-doped region is similar to the exposed substrate region, indicating low loss in the visible range.

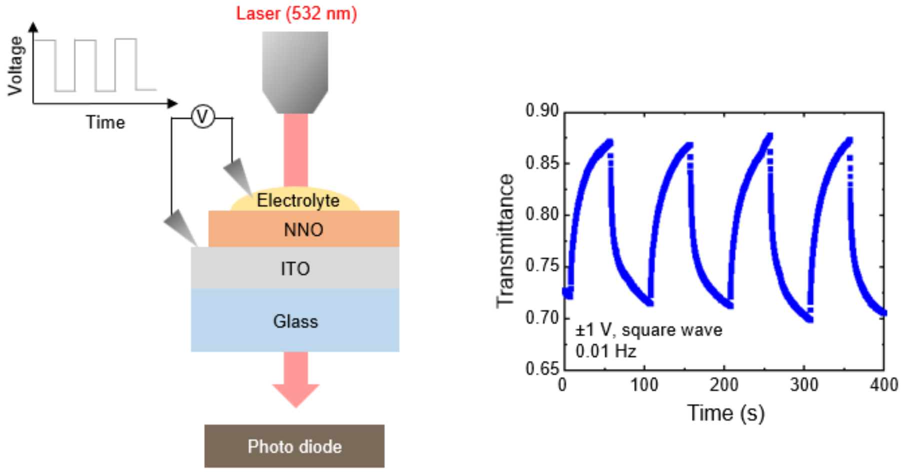

Figure S6. A schematic of our real-time transmittance measurement set-up (left) and data showing reversible transmission modulation of a $NdNiO_3$/ITO glass (right). The electric field was applied in the depth direction of the $NdNiO_3$ film, and a function generator applied ±1 V square wave bias at 0.01 Hz. Salt water was used as the electrolyte to intercalate H ions into the $NdNiO_3$ film. The transmittance modulation data confirms the optical reversibility of the system.

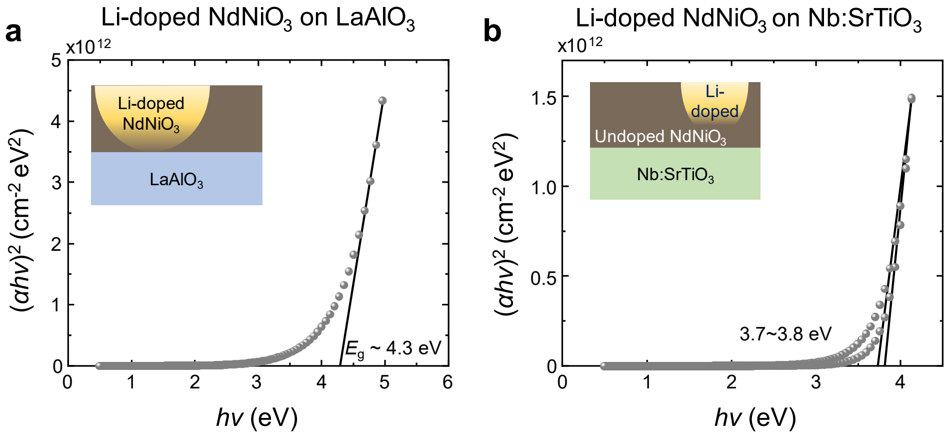

Figure S7. Tauc plots with an assumption that Li-doped $NdNiO_3$ is a direct-gap material. **(a,b)** Tauc plot of Li-doped $NdNiO_3$ on **(a)** $LaAlO_3$ and **(b)** Nb:$SrTiO_3$.

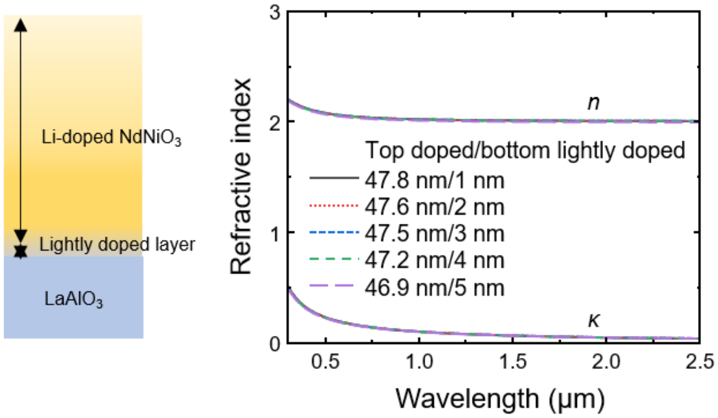

Figure S8. The refractive indices of the Li-doped $NdNiO_3$ on a $LaAlO_3$ substrate obtained from the ellipsometry results (**Figure 3e** in the main text), with different thickness combinations of the top doped and bottom lightly doped $NdNiO_3$. For the ellipsometry data analysis, we enforced the thickness of the bottom lightly doped layer from 1 to 5 nm, with intervals of 1 nm, and therefore, there were three unknown parameters: the refractive index of the top Li-doped and bottom lightly doped layers, and the thickness of the top layer. The refractive index of the Li-doped $NdNiO_3$ is rarely affected by the thickness of the lightly doped layer.

Note S1. Oscillators for the analysis of ellipsometry results

Drude oscillator

The Drude model can be written as:[2]

$$\varepsilon(E) = \varepsilon_1 - i\varepsilon_2 = \varepsilon_{1\infty} - \frac{A_n Br_n}{E^2 - iBr_n E}. \tag{S1}$$

ε_1 and ε_2 are the real and imaginary parts of complex permittivity, ε_∞ is the high-frequency dielectric constant, E is the photon energy in eV, A_n is the oscillator strength of nth oscillator, and Br_n is the broadening factor.

Lorentz oscillator

The Lorentz model can be written as:[2]

$$\varepsilon(E) = \varepsilon_1 - i\varepsilon_2 = \varepsilon_{1\infty} + \frac{A_n E_n}{{E_n}^2 - E^2 + iBr_n E}. \tag{S2}$$

ε_1 and ε_2 are the real and imaginary parts of complex permittivity, ε_∞ is the high-frequency dielectric constant, E_n is the center energy of nth oscillator, E is the photon energy in eV, A_n is the oscillator strength, and Br_n is the broadening factor.

Tauc-Lorentz oscillator

The Tauc-Lorentz model can be written as:[2]

$$\varepsilon(E) = \varepsilon_1 - i\varepsilon_2 = \varepsilon_{1\infty} + \frac{A_n E0_n C_n (E - Eg_n)^2}{(E - {E0_n}^2)^2 + {C_n}^2 E^2} \frac{1}{E} \text{ (for } E > Eg_n). \tag{S3}$$

ε_1 and ε_2 are the real and imaginary parts of complex permittivity, ε_∞ is the high-frequency dielectric constant, A_n is the oscillator strength of nth oscillator, $E0_n$ is the center energy, C_n is the broadening factor, E is the photon energy in eV, and Eg_n is the band gap. If $E < Eg_n$, $\varepsilon_2 = 0$. This model enforces ε_2 to be zero at energies lower than the band gap, Eg.

Gaussian oscillator

The Gaussian model can be given by:[2]

$$\varepsilon_2(E) = A_n/Br_n e^{-(\frac{E-E_n}{\sigma})^2} - A_n/Br_n e^{-(\frac{E+E_n}{\sigma})^2}, \tag{S4}$$

where A_n is the oscillator strength of nth oscillator, E is the energy in eV, E_n is the center energy, and $\sigma = Br_n/2\sqrt{\ln(2)}$.[2] ε_1 can be obtained by Kramers-Kronig relations, which is given by:

$$\varepsilon_1(E) = 1 + \frac{2}{\pi} P \int_0^\infty \frac{E'\varepsilon_2(E')}{E'^2 - E^2} dE', \tag{S5}$$

where P is the principal part of the integration.

Cauchy

Cauchy equation can be given by:[2]

$$n(\lambda) = A_n + \frac{B_n}{\lambda^2} + \frac{C_n}{\lambda^4}. \quad \text{(S6)}$$

A_n represents the long-wavelength index, B_n and C_n are the dispersion terms. This model assumes that the material is lossless, and therefore, $\varepsilon(E) = \varepsilon_1(E) = n^2$.

Two-layer model ($NdNiO_3$ on Nb:STO, Figure 2 in the main text)

The refractive index of the undoped $NdNiO_3$ (on Nb:STO) and the total film thickness (50 nm) were obtained by the ellipsometer measurement on the undoped $NdNiO_3$ region, as shown in **Figure 2b** in the main text. A two-layer model was used to fit the ellipsometer data for the Li-doped $NdNiO_3$ region: the top fully doped $NdNiO_3$ and the bottom undoped $NdNiO_3$ (**Figure 2c**). There are two remaining unknowns at this region: the thickness the Li-doped layer (the thickness of the undoped layer is known because the total thickness of 50 nm was obtained from the undoped region), and the refractive index of the Li-doped $NdNiO_3$. Detailed oscillators are summarized in **Table S1**.

We used one Lorentz oscillator to fit the Li-doped $NdNiO_3$, but we also tried to fit the same data using the Tauc-Lorentz model. The Tauc-Lorentz model enforces ε_2 to be zero—and thus κ = 0—at energies smaller than the band gap (we set the band gap to 3 eV). **Figure S9a** shows the ellipsometry result fitted using the Lorentz model, and **Figure S9b** shows the same result fitted using the Tauc-Lorentz model. Both fits seem to be almost identical, with similar thicknesses. However, the Lorentz model has a slightly better fit to the experimental data: compare Δ at 60 deg (red) at the wavelength of 0.3 μm, for both cases. The Lorentz model follows the abrupt increase at 0.3 μm, whereas the Tauc-Lorentz model does not. The refractive indices obtained by both models are very similar at wavelengths longer than ~0.5 μm. However, the imaginary part of the refractive index (κ) fitted by the Tauc-Lorentz model (**Figure S9d**) approaches zero at ~0.4 μm, which corresponds to the band gap (3 eV).

Note that although we divided our NdNiO3 sample into two layers—a fully doped layer and an undoped layer—there is likely also a very thin but non-negligible intermediately doped region between the two layers that we didn't explicitly consider. Accordingly, our measured refractive index represents an effective refractive index that includes contributions from both fully and partially doped NdNiO3. In this case, the partially doped part would have a small but non-zero κ even at energies lower than the band gap."

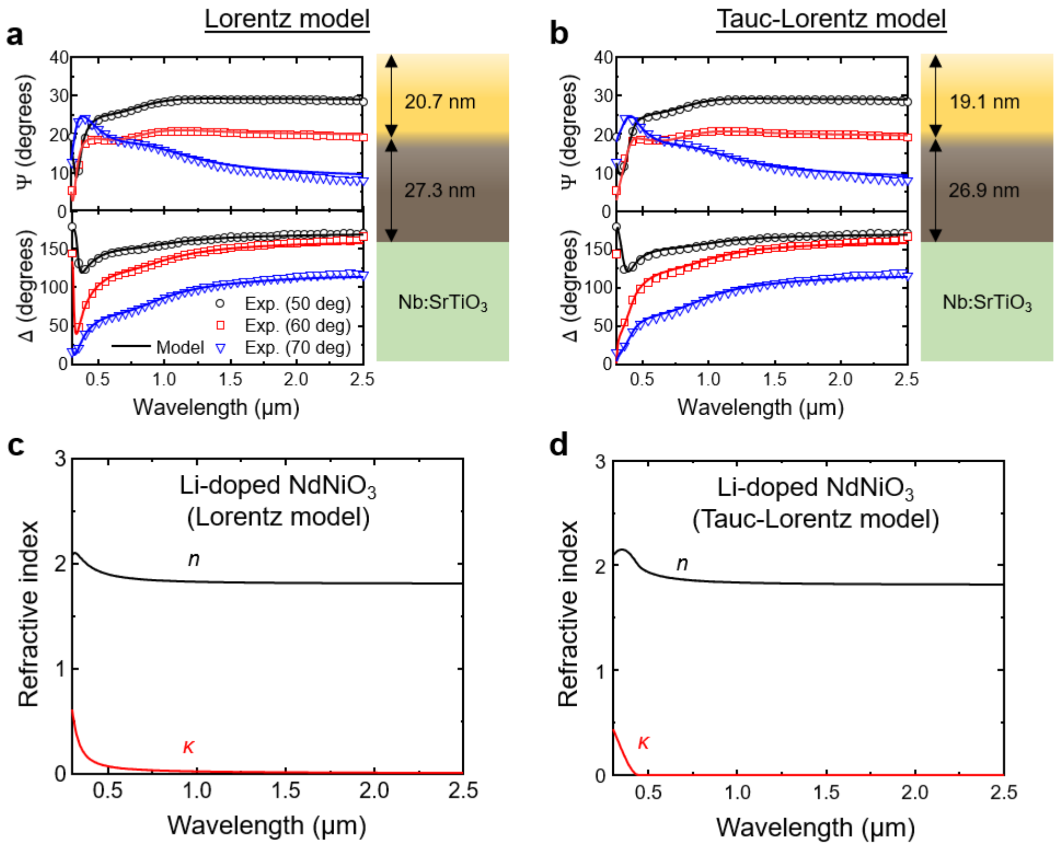

Figure R9. Comparison of the ellipsometry measurements of the Li-doped $NdNiO_3$ on Nb:STO fitted using the Lorentz model and the Tauc-Lorentz model. **(a, b)** The ellipsometry ψ and Δ, and **(c, d)** the refractive index obtained from each model.

Two-layer model ($NdNiO_3$ on $LaAlO_3$, Figure 3 in the main text)

Based on the ToF-SIMS measurement (**Figure 3f** in the main text) and the degree of visible transparency we observe, we assumed that the thickness of the bottom lightly doped layer is most likely smaller than 5 nm. We then performed 5 separate fits, each assuming a different thickness of the bottom layer from 1 to 5 nm. Each of these fits had three fitting parameters: the refractive index of the top and bottom $NdNiO_3$ layers, and the thickness of the top Li-doped layer. We then found the best of the 5 fits, with the result shown in **Figure 3e**. The estimated thickness in this region (47.5 + 3 nm = 50.5 nm) almost matches the overall thickness of 52 nm. Refer to **Figure S8** for the refractive index values of the top doped $NdNiO_3$ layer obtained by the five separate fits.

Table S1. Summary of oscillator types and values used in our work.

Samples	#	Oscillators	A_n (eV)	E_n (eV)	Br_n (eV)
**$NdNiO_3$ on Nb:STO**					
**Substrate (Nb:STO)**			$\varepsilon_{1\infty}$ = 2.1281		
	1	Drude	1.6942		0.21693
	2	Gaussian	0.13904	2.2568	1.878
	3	Gaussian	2.6945	4.0872	0.52314
	4	Gaussian	5.1596	4.7317	1.1368
	5	Gaussian	4.5801	6.4149	0.21157
Undoped $NdNiO_3$			$\varepsilon_{1\infty}$ = 2.8872		
	1	Drude	9.9825		1.0518
	2	Lorentz	5.1795	5.2621	1.1724
	3	Lorentz	5.1123	2.7884	2.6075
	4	Lorentz	2.8448	1.1441	1.0536
	1	Lorentz	13.23	7.259	5.9142
Doped $NdNiO_3$ (120 s)			$\varepsilon_{1\infty}$ = 2.3774		
	1	Lorentz	4.3692	4.4246	1.1855
Doped $NdNiO_3$ (180 s)			$\varepsilon_{1\infty}$ = 2.6308		
	1	Lorentz	3.3265	4.3961	1.0661
$NdNiO_3$ on LAO					
Substrate (LAO)			$\varepsilon_{1\infty}$ = 3.9574		
	1	Cauchy	A_n = 0.39829, B_n = 0.043615 (these values are dimensionless)		
	2	Lorentz	0.35047	0.02257 (182.0 cm^{-1})	0.33821 x 10^{-3}
	3	Lorentz	0.22239	0.05287 (426.4 cm^{-1})	0.55318 x 10^{-3}
	4	Lorentz	0.22878 x 10^{-3}	0.06148 (495.8 cm^{-1})	0.47724 x 10^{-3}
	5	Lorentz	0.022063	0.08073 (651.1 cm^{-1})	2.4718 x 10^{-3}
	6	Lorentz	4.0938 x 10^{-3}	0.08446 (681.1 cm^{-1})	4.4059 x 10^{-3}
Undoped $NdNiO_3$			$\varepsilon_{1\infty}$ = 1.8425		
	1	Drude	10.092		0.9752
	2	Lorentz	13.32	6.8453	2.6173
	3	Lorentz	3.7493	2.6041	2.1559
	4	Lorentz	2.8842	1.1241	1.0388
Doped $NdNiO_3$			$\varepsilon_{1\infty}$ = 1.0729		
	1	Lorentz	4.8415	6.3222	3.7405

Lightly doped layer	$\varepsilon_{1\infty}$ = 9.3364				
	1	Lorentz	95.653	0.94989	10

Note S2. Time-of-flight (ToF) secondary ion mass spectrometry (SIMS)

An Ar^+ beam (20 kV, 500 nA) was used for milling of an area of 1 mm × 1 mm over a sample, and a Bi^+ beam (30 kV) was used for the primary analysis over a 100 µm × 100 µm zone located at the center of the milled area. During the measurements, a low-energy electron gun and a gas gun (Ar^+) were used to neutralize the surface charge.

Supplementary References

[1] U. Sidik, et al., "Tunable Proton Diffusion in NdNiO3 Thin Films under Regulated Lattice Strains," *ACS Applied Electronic Materials*, vol. 4, no. 10, pp. 4849-4856, 2022, https://pubs.acs.org/doi/10.1021/acsaelm.2c00711.

[2] H. Fujiwara and R. W. Collins, *Spectroscopic ellipsometry for photovoltaics*, Springer, Cham, 2018, https://doi.org/10.1007/978-3-319-75377-5.
